# Obscure Nervous Diseases Popularly Explained

**Published:** 1856-10-01

**Authors:** 


*Obscure Nervous Diseases popularly explained,* in Six Letters to a Physician on the many Nervous Affections resulting from Dental Irritation and other sources of Reflex Nervous Disturbance. By J. L. Levison. Dedicated, by permission, to Dr. Conolly. London: Effingham Wilson, Royal Exchange. 1856.

When any attempt is made to render our knowledge of the nervous system more satisfactory, or to explain the *morbific* causes of some of the anomalous forms of nervous diseases which particularly affect civilized man, the person who thus enlightens us is entitled to our best thanks. The latter Mr. Levison has done. The work before us is worthy the attention of medical men, although it is written in a clear and popular style, and nearly divested of all technical phraseology; and yet its physiology and doctrine of diseased conditions are strictly scientific.

Mr. Levison practised many years as a surgeon-dentist, and applied himself with great ardour and zeal to the elucidation of the nervous affections, more or less complicated, which were induced by dental irritation, and also others resulting from disturbing influences of another kind: and he has brought some addition to the existing knowledge by tracing the tissues directly or indirectly implicated, demonstrating in the clearest manner that dead stumps, polypi at the fangs, &c., may affect the brain, nervous systems, the heart, lungs, stomach, &c.; and cites cases where extensive disturbance of these organs has occurred, when there did not appear any pain or uneasy sensation in the mouth—the actual seat of the primary disturbance—and yet, after years of suffering, the nervous affection had been mitigated or cured by the removal of the offending and irritating bodies.

We must, from want of space, just glance at the contents of these letters.

The first letter enters into an examination as to what constitutes a normal man, and that barbaric races give undue attention to physical training, and civilized man too little; thus, by over mental stimulation, so much injury results.

The second letter deprecates the discrepant and contradictory treatments of some forms of neuralgia; and he then unfolds his views as to how to form a correct *diagnosis* in such anomalous diseases.

The third letter explains the sympathy of the stomach, alimentary canal, &c., with the buccal cavity.

The fourth letter clearly explains the nervous connexion of the teeth (through the fifth pair of nerves) with the eyes, ears, nostrils, glands, fauces, &c., and importance of this knowledge in removing much of human suffering.

The fifth letter is highly important: it treats of dental irritation inducing affections of the muscles of the face, neck, &c., and often paralysis and catalepsy. In this communication Mr. Levison has also discussed what he calls *simulated* affections, resembling hysteria, epilepsy, hemicrania, and temporary insanity, and clearly points out how to distinguish the *pseudo* from the ordinary forms of such affections.

The sixth letter contains also much valuable information on sedentary habits, over-anxiety, too much mental application, &c. &c.

Mr. Levison has, to use a legal term, made out a case, and has established an important fact, that it is impossible to arrive at a knowledge of the cause of many anomalous forms of nervous diseases without taking the organs of the mouth into consideration; and we strongly recommend a perusal of this small but unostentatious work.

